# Clinical Lectures on a Case of Diabetes, Delivered at St. Mary's Hospital

**Published:** 1865-01

**Authors:** Thomas K. Chambers

**Affiliations:** Lecturer on Clinical and Systematic Medicine at, and Physician to the Hospital.


*Clinical Lecture on a Case of Diabetes.* Delivered at St. Mary's Hospital, by THOMAS K. CHAMBERS, M. D., Lecturer on Clinical and Systematic Medicine at, and Physician to the Hospital.

William S——, aged twenty-two, a thin, young-looking, farm-laborer, has suffered for at least two years with ailments of various kinds, which are commonly considered by pathologists to be symptomatic of diabetes. He has been weak and unable to work, [illegible]t always thirsty and usually hungry, and passed a large quantity of urine. For the last year he has been unable to work at all.

He was admitted into St. Mary's, May 31st. At this time the symptoms I have named were noticed. The chest was examined and found healthy, the pulse was slow and regular, and the bowels opened daily. The skin was normally moist, and he stated that at night he often perspired. His sleep was sound, except when he was roused by his bladder getting full of urine. His weight was 95½ pounds. He remained in the hospital five weeks, during which period the following variations in the urinary symptoms, with the changes in weight during the corresponding time, and the alterations in treatment to which I trace these changes, are noted in a table condensed from the case-book:

During the *first* week the total quantity of urine passed was 680 fluid ounces.

Of the specific gravity before fermentation, 1,042.

" " " after " 1,012.

His weight had increased to 96 pounds.

During the *second* week the total quantity of urine passed was 449 fluid ounces.

Of the specific gravity before fermentation 1,039 to 1,040.

His weight had decreased to 95½ pounds.

During the *third* week the total quantity of urine passed was 472 fluid ounces.

Of the specific gravity before fermentation, 1,039 to 1,041.

His weight had increased to 97 pounds.

During the *fourth* week the total quantity of urine passed was 452 fluid ounces.

Of the specific gravity before fermentation, 1,040 to 1,042.

His weight had increased to 97½ pounds.

During the *fifth* week he lost half a pound in weight, and then left the hospital by his own desire.

During the *first* week he was treated with a grain of opium every night, a mutton chop for breakfast, in addition to "full diet," with three captain's biscuits, in place of bread, daily.

During the *second* week the opium was left off, and the treatment altered to eight grains of iodide of potassium, three times a day, with a drachm of cod-liver oil; he was allowed but one captain's biscuit, instead of bread, daily, but as much meat as he could eat, and as much milk as he could drink.

During the *third* week the captain's biscuit was changed to bran biscuit; but in point of fact he did not eat that substance, preferring to go without breadstuffs altogether. No other change was made.

During the *fourth* week no change was ordered in the treatment.

In the *fifth* week an attempt was made to get him to eat Van Abbott's gluten bread toasted and buttered, but in vain.

The first practical point to observe in the pathology of diabetes mellitus is the arrest in the function of constructive growth. That generally used material of nutrition, sugar, which ought to be assimilated as food, and made available to the growth of the body, passes in and out again of the thoroughfare of the circulation unaltered, and is ejected in the urine. And here I refer, not only to the sugar, which is taken as such into the mouth, but also to that which is formed out of starch, by the action of the saliva. So that in a thorough diabetic, the whole of the saccharine and amylaceous matters in the dietary are utterly wasted. Trying to feed him upon them would be just the same as feeding him upon nothing at all. More than this—I think it would be doing him harm.—These useless articles of food, though they contribute nothing to his support, stop his appetite, and so he does not eat the needful quantity of really nourishing things; and, moreover, the analogy of other diseases would lead to the conclusion that the burdening a disabled function with work to which it is unequal, will disable it more and more. If the stomach rejects undigested an ounce of beef, it is made worse by the administration of a steak. If the eye-sight fails or the brain reels on slight exertion, common experience prevents us from demanding violent efforts.

Therefore, you find that cutting off a patient's sugar, bread and potatoes by no means lowers him. On the contrary, he often gets heavier under the restriction. And one can easily believe the instances recorded by Dr. Pavy, where treacle, honey and sugar, intentionally administered as an experiment to diabetics, made the patients feel worse, and lose weight. I do not mention in evidence, or assign any importance to the increase or diminution of sugar in the excretions under the influence of saccharine or non-saccharine diet. It is less when little starch and sugar is taken; it is more when much is taken. But the real point is the acquirement of flesh; and the test, the addition of weight. You will find, when the ordinary mixed diet is used by diabetics, that much flesh is lost; and that it is regained when a carniverous dietary is rigidly enforced upon them. With the flesh also comes strength, showing that muscle is gained, and not mere fat.

The great point, then, in the treatment of diabetes is to accustom the patient gradually to live entirely on meat, or, at least, entirely on albuminous and glutinous food. This need not seem a mighty hardship; the iron-framed Esquimeaux do it, and the wiry, tough, half-breeds of the Pampas, with a bill of fare certainly less varied in flesh-meat than our European meadows afford. You may, then, fairly direct your energies to attain this goal with a good chance of success. What nations live and increase upon may be trusted to nourish a single individual.

Laying this down as the main point in the treatment, let us see what is likely to be gained by it.

You will learn from the history of the present patient, that turning him into a carniverous animal does not entirely remove a diabetic's peculiar aliment. Twenty days after all vegetable matter had been cut off from his diet card, and he had been carefully watched by others set to detect any infringement of rules, still the urine is full of sugar. So that it must be derived from some other quarter than the starchy and saccharine constituents of the food. We shall feel less surprise at this formation of sugar from animal matter alone when we call to mind that there is even a normal secretion in which it may be found under even normal circumstances. The milk of carnisora contains it.*

Moreover, sugar may be found in the laboratory by a process of decomposition without the presence of life. Nay, rather, only when life is extinct. The simple application of oxygen will cause some animal substances to be converted into sugar. This has been noticed by Dr. Claude Bernard to be especially the case with the tissues which form the secreting substance of the liver, which, carefully washed from blood and exposed to the air, quickly becomes copiously saccharine. So that your patient has a fertile source in his own body, even if none of the other parts possess the same property. He carries about him two pounds of viscus capable of easy conversion into sugar.

But remark, it is *dead* liver, not *live* liver, which in health is decomposed as above stated. Normal vital action seems to have got another way of removing the hepatic substance, for during life no sugar can be detected as formed from this organ. Diabetes, then, like all diseases of which we know more than the superficial symptoms, turns out to be a death in life—an anticipation of the post-mortal properties of the bodily constituents. This is an additional reason for casting about how best to apply restorative medicine in the treatment, and for urging an ample supply of material for revivifying the frame. If the dying liver is passing off quickly by the kidneys, we must quickly give the patient the wherewithal to make new liver. Now you gain an additional reason for enforcing animal diet in diabetes.

To accustom this patient to leave off by degrees vegetable aliments, I allowed him first captain's biscuit for a fortnight. During that time scarce any weight was gained, and the urine was but little altered. He liked those biscuits very well. I then ordered him bran biscuits, but he said they were so nasty he could not eat them, and he wasted some of his milk in trying to make them palatable. However, he increased in weight by two pounds during two weeks, and made eleven pints less urine weekly than on his first admission; and this, although he drank as much fluid as he felt disposed to take.

*Bensch has put on record the presence of sugar in the milk of bitches fed entirely on meat.

After this Mr. Van Abbot was good enough to give him a supply of the gluten bread sold by that firm. For a week he tried hard to eat it in addition to his former allowance of meat; but I am sorry to say he failed in acquiring a taste for it. His appetite fell off during the experiment; he lost half a pound of the weight he had acquired, and was so annoyed at being pressed to eat the gluten bread that he insisted on returning home on July 12th.

My own feeling is, that we do not act wisely in enforcing a dietary which is really unbearable by the patient in any chronic disease. The great object to be gained is to conciliate the stomach, appetite and fancy, into taking the greatest possible amount of animal food; and if practically you find that the patient eats more by having a biscuit or a crust, or even vegetables with his meals, it is better to allow it him than to act the tyrant.

As to drugs, opium was given to this patient for a week. It did not seem to exercise any influence at all. However, in some cases, it certainly does seem to diminish the excretion of urine, for the amount did not increase again after it was left off. But is that any advantage? or is it only from our education in allopathic prejudices that we reckon on help from such interference? I confess it seems to me that if the blood gets loaded with sugar, as analysis proves that it does, it is better that it should be washed out by an ample diuresis, than that it should remain at the risk of poisoning the tissues. I have never distinctly traced any harm to opium, truly; but I have traced harm to a drug whose action is similar. Cinchona also diminishes the flow of urine; and I once gave that to a diabetic patient. After a short time he got comatose, and died with effusion in the ventricles. The effused serum was loaded with sugar, which I suppose it was the business of diuresis to have washed away.

For this reason I avoid cinchona in diabetes, even when I desire to give tonics for the sake of increasing the appetite. I prefer iron and strychnine. An elderly patient of mine, with moderate diabetes, is now taking those drugs with advantage to his strength and digestion, and without any deleterious action on the kidneys.

The iodide of potassium which you see prescribed on his medicine card was given on purely empirical grounds. There are no drugs known to do good to the essential phenomena of diabetes; there were no secondary symptoms demanding special medication, so I thought it a fair case for an experiment. The result was, that at all events no harm was done; the patient continued to increase in weight and strength, and did not exhibit any of the usual symptoms of intoxication by iodine.

This is not like substituting an experiment in search of a possible specific in place of rational treatment; such conduct is, indeed, most reprehensible. But here there is no medicine omitted, for there is none to be given that offers any hope of its possessing an alterative agency, and it is a question between either something new or a mere placebo. I shall try the iodide of potassium again on the next pure and uncomplicated case.

People sometimes feel a doubt how far they ought to gratify the patient's unnatural thirst. On this point the same considerations weigh with me which influence my objections to cinchona—I think there ought to be kept up a flow of water through the system in proportion to the abnormal quantity of sugar in the blood, in order that no retention or discharge in unusual places of this material may take place. I therefore let patients drink as much as they feel disposed.

You will find that the demand for drink is closely proportioned to the quantity of sugar required to be got rid of. Thus, when the dietary is changed from starchy to meat food, much less fluid is drunk and much less is evacuated by the kidneys, though no restriction is placed upon the thirst. Such was the case with the lad now lectured upon; during the second week he made twelve pints less urine, though he was recommended to drink as much water as he liked. The specific gravity, also, of the secretion was not raised, which it certainly would have been had the diminution in quantity depended on any other cause than the diminution of the instinctive call for diluents. I believe the excessive thirst depends on the saccharine contents of the blood; it is, therefore, wise to gratify it, and provide the normal outlet for the abnormal constituent.

